# Serum Neutralization of SARS-CoV-2 Omicron BA.1 and BA.2 after BNT162b2 Booster Vaccination

**DOI:** 10.3201/eid2806.220503

**Published:** 2022-06

**Authors:** Rune M. Pedersen, Line L. Bang, Lone W. Madsen, Thomas V. Sydenham, Isik S. Johansen, Thøger G. Jensen, Ulrik S. Justesen, Thomas E. Andersen

**Affiliations:** Authors affiliation: University of Southern Denmark, Odense, Denmark

**Keywords:** COVID-19, respiratory infections, severe acute respiratory syndrome coronavirus 2, SARS-CoV-2, SARS, coronavirus disease, zoonoses, viruses, coronavirus, serum neutralization, Omicron, BA.1, BA.2, Denmark

## Abstract

The SARS-CoV-2 Omicron variant BA.2 sublineage is rapidly replacing earlier Omicron lineages, suggesting BA.2 has increased vaccine evasion properties. We measured neutralization titers of authentic BA.1 and BA.2 isolates in serum samples from persons who received the BNT162b2 booster vaccine. All samples neutralized BA.1 and BA.2 at equal median values.

The emergence of the SARS-CoV-2 Omicron variant BA.1 (B.1.1.529) in late 2021 caused immediate apprehension because it readily outcompeted the already highly transmissible Delta variant. The high number of spike mutations and indications of substantial vaccine evasion properties prompted the World Health Organization to designate BA.1 a variant of concern on November 26, 2021 (*1*). Recently, a large-scale epidemiologic study addressed concerns about BA.1 vaccine evasion by showing that 2-dose vaccination and an mRNA vaccine booster dose provides at least temporary protection against this variant (*2*).

Surveillance data from countries where Omicron was initially detected indicate that BA.1 is a transient lineage that is rapidly replaced by the Omicron sublineage BA.2 (D. Yamasoba et al., unpub data, https://doi.org/10.1101/2022.02.14.480335). By now, the BA.2 sublineage is dominant in >18 countries and is progressing in the United States, where cases are doubling weekly (*3*). The higher transmissibility of this sublineage in countries with high vaccination rates could indicate that BA.2 escapes vaccines even better than BA.1.

We investigated BA.2 vaccine escape in vitro by directly comparing the neutralization of authentic BA.1 and BA.2 strains in serum samples from persons who had received 2 doses of the BNT162b2 Pfizer-BioNTech (https://www.pfizer.com) mRNA vaccine and 1 booster dose. We analyzed serum samples collected during January 26–28, 2022, from 20 (8 male, 12 female) immunocompetent SARS-CoV-2–naive participants by using a 90% plaque reduction neutralization test (PRNT_90_), as previously described (*4*). Median age among participants was 57 years (interquartile range [IQR] 50–60 years). We performed whole-genome sequencing by using a MinION (Oxford Nanopore Technologies, https://nanoporetech.com) sequencing instrument to identify the clinical isolates used for PRNT_90_. We obtained lineage and mutation calls by using Pangolin version 3.1.17 with pangoLEARN database 06–12–2021 and Nextclade version 0.14.4 (*5*,*6*). Viral genome sequences are available on GenBank (accession nos. ON055855 for the ancestral strain, ON055874 for BA.1, and ON055857 for BA.2). We analyzed all serum samples for antibodies by using the Liaison TrimericS IgG Quantitative immunoassay (DiaSorin, https://www.diasorin.com). Participants signed informed consent forms, and the study was approved by the Regional Committees on Health Research Ethics for Southern Denmark (no. S-20210007C). Experiments with live SARS-CoV-2 were conducted in approved Biosafety Level 3 facilities (license no. 20200016905/5).

Among vaccinated participants, the median time between the first and second BNT162b2 dose was 35 (IQR 34–36) days; median time between the second and booster doses was 168 (IQR 154–174) days. The median time between the booster dose and serum sampling was 42 (IQR 40–42) days. The median PRNT_90_ toward the ancestral SARS-CoV-2 strain was 320 (IQR 160–480), whereas the median PRNT_90_ toward BA.1 and BA.2 was 40 (IQR 20–80) for both. All neutralization titers measured for BA.1 and BA.2 were above the threshold limit (Figure). Using the Wilcoxon signed-rank test, we assessed differences between the neutralization titers of the ancestral strain and the 2 Omicron strains and found statistically significant differences for both strains (BA.1, p<0.0001; BA.2, p<0.0001), whereas the difference between BA.1 and BA.2 was not statistically significant (p = 0.1953). The median antibody level measured by Liaison immunoassay was 4,115 (IQR 2,675–6,000) binding antibody units/mL. We used the Spearman coefficient (ρ) to correlate the neutralization titers toward the ancestral strain (ρ 0.8073; p<0.0001), BA.1 (ρ 0.7396; p = 0.0002), and BA.2 (ρ 0.7496; p = 0.0002) (Appendix Figure).

**Figure:**
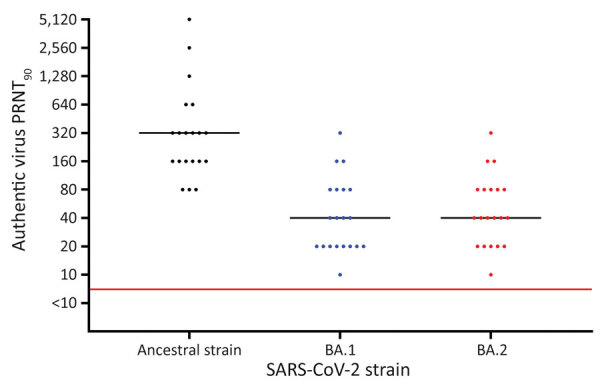
Results of PRNT_90_ of serum against SARS-CoV-2 ancestral strain and Omicron sublineages BA.1 and BA.2 after BNT162b2 (Pfizer-BioNTech, https://www.pfizer.com) booster vaccination, Denmark. Serum samples were collected from 20 SARS-CoV-2–naive participants who received 2 BNT162b2 doses and a booster BNT162b2 dose. Viral genome sequences are available in GenBank (accession nos. ON055855 for the ancestral strain, ON055874 for BA.1, and ON055857 for BA.2). Red line indicates neutralization threshold; black lines indicate median neutralization titers for each strain. PRNT_90_, 90% plaque reduction neutralization test.

Although vaccine evasion appears to be a key feature of both BA.1 and the emerging BA.2 strain, according to our results, BA.2 does not evade the humoral immune response induced by the BNT162b2 vaccine better than BA.1 does. Thus, the current surge of BA.2 seems to occur as a result of mechanisms of transmissibility other than antibody escape.

At the time of this submission, J. Yu et al. (*7*) had published data from serum neutralization of lentivirus constructs that express the spike proteins of BA.1 and BA.2. Our authentic virus results are in accordance with theirs; Yu et al. found that BA.1 and BA.2 pseudoviruses were neutralized equally and at median values ≈8 times lower than the ancestral strain. In addition, our PRNT_90_ results showed that all serum samples neutralized the Omicron variants at or above the neutralization threshold value of 10 at the 6-week post-booster time point (Figure). This finding is in accordance with the epidemiologic results on BA.1–specific vaccine effectiveness reported by N. Andrews et al. (*2*), which showed that 2 doses and a booster of BNT162b2 provides a certain degree of protection against symptomatic BA.1 infection. Assuming that the neutralization data of BA.2 also reflects vaccine effectiveness, our results indicate that at 6 weeks after a booster dose persons should at least be temporarily protected against mild disease with this sublineage. Health agencies should continue to encourage booster vaccination for persons who have received 2 doses of BNT162b2.

Appendix Additional information on serum neutralization of SARS-CoV-2 Omicron BA.1 and BA.2 after BNT162b2 booster vaccination in relation to serum antibody levels.
