# Supplementary material for: Serum Neutralization of SARS-CoV-2 Omicron BA.1 and BA.2 after BNT162b2 Booster Vaccination
Source: Emerg Infect Dis. 2022 Jun;28(6):1274–5. doi: 10.3201/eid2806.220503 (PMC9155893; doi:10.3201/eid2806.220503)
Supplement: Appendix — Additional information on serum neutralization of SARS-CoV-2 Omicron BA.1 and BA.2 after BNT162b2 booster vaccination in relation to serum antibody levels. [file 22-0503-Techapp-s1.pdf]

Article DOI: https://doi.org/10.3201/eid2806.220503

Serum Neutralization of SARS-CoV-2 Omicron BA.1 and BA.2 after BNT162b2 Booster Vaccination

Appendix

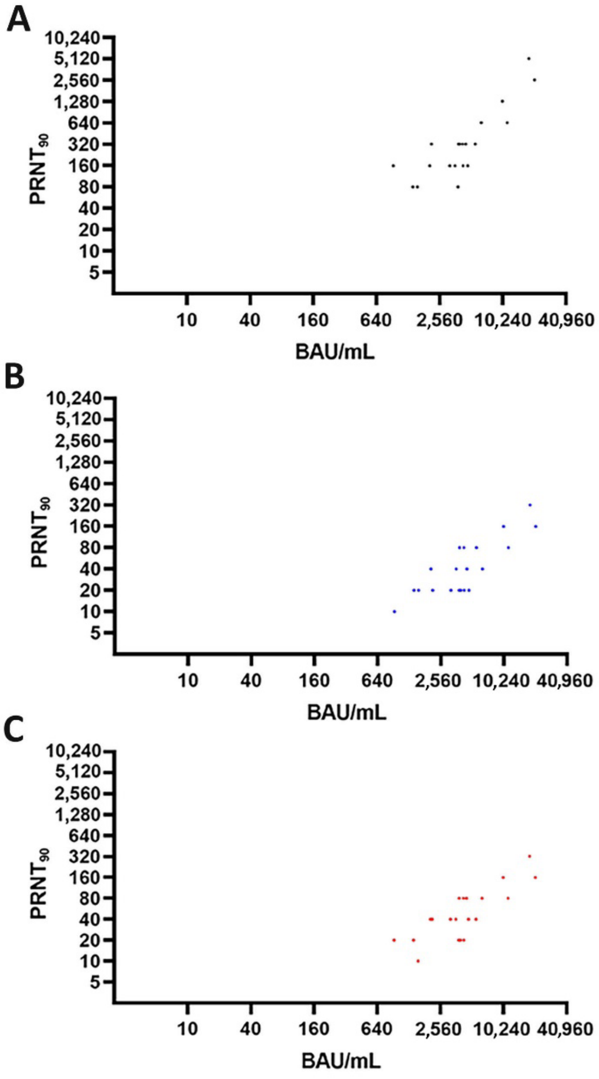

Appendix Figure. Results of 90% plaque reduction neutralization tests ($PRNT_{90}$) against 3 SARS-CoV-2 strains in relation to binding antibody unit per mL (BAU/mL). Serum samples were collected from 20 SARS-CoV-2–naive participants who received 2 BNT162b2 (Pfizer-BionTech; Pfizer,

https://www.pfizer.com) doses and a booster BNT162b2 dose. Titers were measured by using Liaison TrimericS IgG Quantitative immunoassay (DiaSorin, https://www.diasorin.com). A) $PRNT_{90}$ titers of the ancestral SARS-CoV-2 strain (GenBank [https://www.ncbi.nlm.nih.gov/genbank] accession no. ON055855). B) $PRNT_{90}$ titers of the BA.1 SARS-CoV-2 strain (GenBank accession no. ON055874). C) $PRNT_{90}$ titers of the BA.2 SARS-CoV-2 strain (GenBank accession no. ON055857).
